# Coarse-Grained Models for Protein-Cell Membrane Interactions

**DOI:** 10.3390/polym5030890

**Published:** 2013

**Authors:** Ryan Bradley, Ravi Radhakrishnan

**Affiliations:** 1 Department of Chemical and Biomolecular Engineering, University of Pennsylvania, Philadelphia, PA 19104, USA; 2 Department of Bioengineering, University of Pennsylvania, Philadelphia, PA 19104, USA

**Keywords:** molecular dynamics, coarse-grained model, membrane proteins

## Abstract

The physiological properties of biological soft matter are the product of collective interactions, which span many time and length scales. Recent computational modeling efforts have helped illuminate experiments that characterize the ways in which proteins modulate membrane physics. Linking these models across time and length scales in a multiscale model explains how atomistic information propagates to larger scales. This paper reviews continuum modeling and coarse-grained molecular dynamics methods, which connect atomistic simulations and single-molecule experiments with the observed microscopic or mesoscale properties of soft-matter systems essential to our understanding of cells, particularly those involved in sculpting and remodeling cell membranes.

## 1. Introduction

In more than three decades since the first molecular dynamics simulation of a protein [1], molecular dynamics methods have emerged as an effective tool for simulating biological soft matter, thanks to the careful development of force fields and simulation methods. The synthesis of models for soft-matter physics and protein dynamics provides great insight into a wide range of biological processes important to understanding human health.

**Figure 1:**
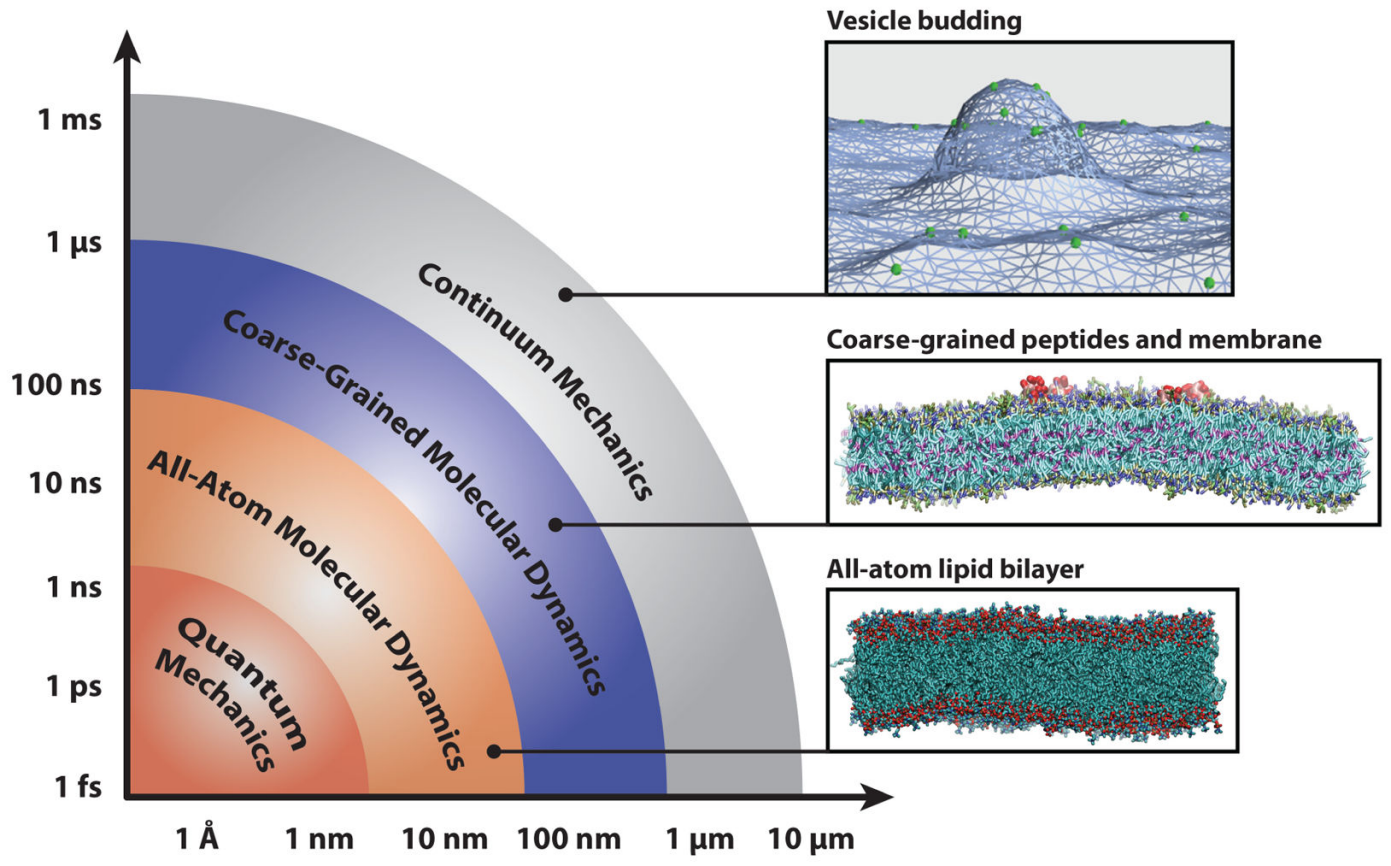
Diagram of computational methods for studying biophysical systems across a range of time- and length-scales. Representative snapshots depict an all-atom lipid bilayer, peptides embedded in a coarse-grained bilayer and proteins remodeling a continuum mechanics membrane model. Bilayers were simulated with the CHARMM36 [15] and Martini [16] force fields and rendered with Visual Molecular Dynamics [17].

Molecular dynamics simulations at both atomistic and coarser levels of detail allow us to probe the properties of complex biomolecular systems with numerical methods. While molecular dynamics simulations collapse many degrees of freedom into relatively few, they are nevertheless capable of reproducing a host of important physical phenomena that result from the collective action of complex particles. In addition to separate applications in soft matter and protein systems, many simulation studies have investigated the crucial interactions between lipid bilayers and the proteins that remodel them. These interactions are crucial to a wide range of cellular processes, including membrane remodeling in endocytosis [2], the action of protein-gated ion channels [3–5], the assembly of membrane proteins [6–9], mediation of membrane fusion [10–12], and the activation of membrane-protein-based signaling networks [13,14]. The interactions of proteins with lipid bilayers are vital to our understanding of these phenomena; this necessitates the use of models that span several time- and length-scales (delineated in Figure 1), as well as careful matching to experimental results.

Simulations have become more powerful in recent years, thanks to a combination of increased computer power, advanced sampling methods, distributed computing and specialized hardware [18–20]. However, it is the parameterization of force fields that are capable of matching experimental data at multiple length scales, from X-ray scattering data to protein crystal structures, that makes these models into useful microscopes for studying cell systems.

**Figure 2:**
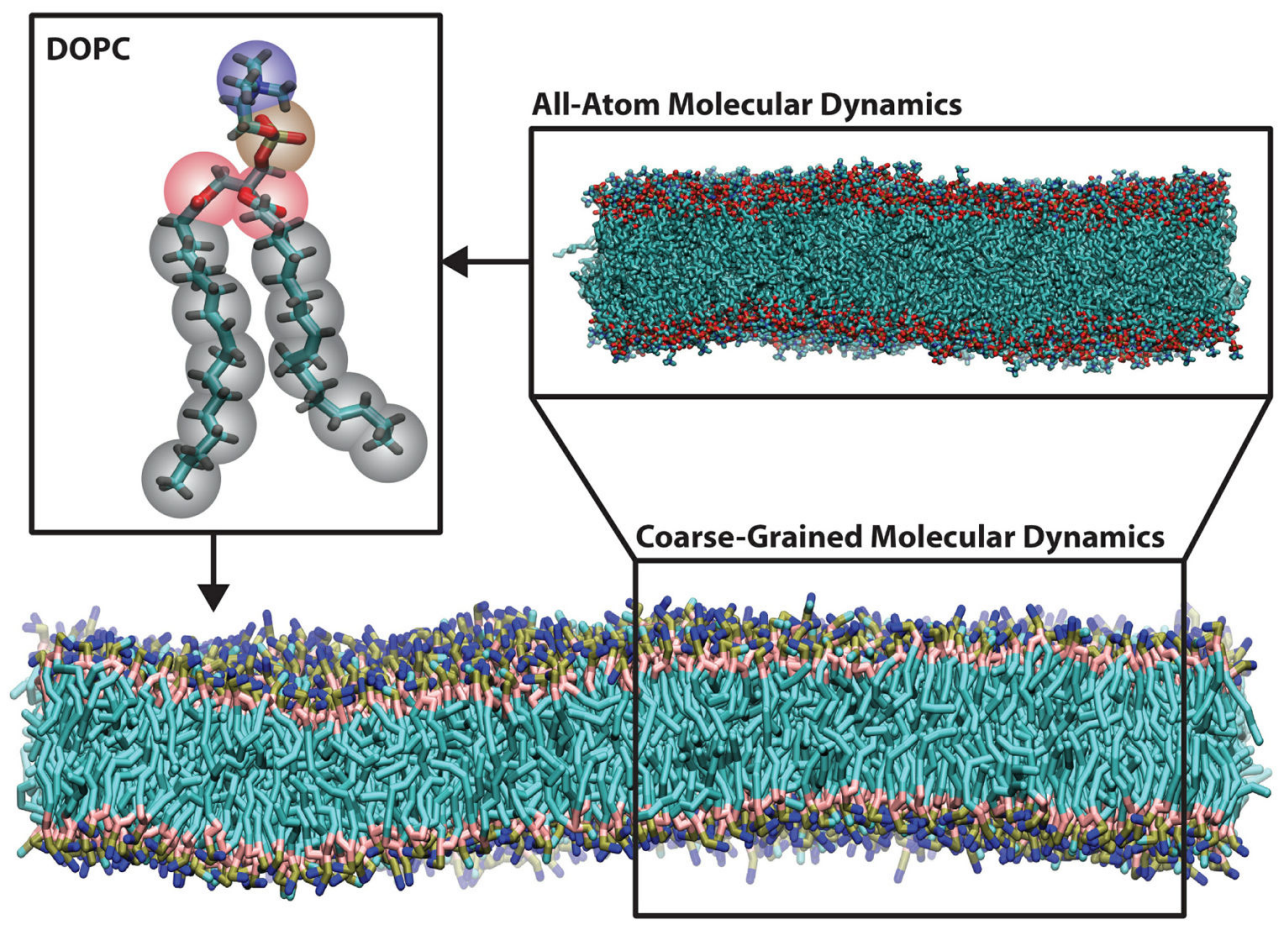
Representative snapshots of all-atom (upper right) and Martini coarse-grained (bottom) molecular dynamics simulations of a 4:1 dioleoylphosphatidylcholine with dioleoylphospatidylserine (DOPC/DOPS) bilayer. The upper left shows the coarse-grained mapping of a single DOPC lipid, with beads colored by bead type (gray for hydrocarbons-, pink for glycerol-, brown for phosphate- and blue for choline-type). The all-atom system contains 800 lipids, while the coarse-grained system contains 3,200 lipids (water molecules are not pictured here). Bilayers were simulated with the CHARMM36 [15] and Martini [16] force fields and rendered with Visual Molecular Dynamics [17].

Coarse-grained molecular dynamics simulations employ intermediate resolution in order to balance chemical detail with system size, see Figure 2. They offer sufficient size to study membrane-remodeling events, while retaining the ability to self-assemble. Because they are capable of simulating mesoscopic length scales, they make contact with a wider variety of experiments, many of which lack the precision to easily inform small atomistic models.

A complete coarse-grained model must include two components: a mapping from atomistic structures to coarse-grained (CG) “beads” and a set of potentials that describe the interactions between beads. The former defines the geometry or length scale of the resulting model, while the latter defines the “force field”. The parameterization of the force field is essential to the performance of the model, which is only relevant insofar as it can reproduce experimental observables. Recent improvements to widely-disseminated force fields have strengthened both their transferability—the ability to use a model on a novel biophysical system with straightforward parameterization—as well as tunability, the ability to customize a model to match a desired quantity [21–23].

In this review, we will describe the characteristic methods for developing coarse-grained molecular dynamics (CGMD) models, namely, the “bottom-up” force-matching- and “top-down” free energy-based approaches. We will illustrate the myriad ways in which these models can reproduce protein dynamics, bilayer physics and experiments that probe protein-membrane interactions. We will survey the applications of coarse-grained models to protein-membrane interactions and describe the ways in which CGMD simulations make contact with experiments and simulations at larger and smaller length scales. We note that excellent reviews have been written on coarse-grained methods with applications in other fields, such as polymer physics; see, e.g., the recent review by Li *et al.* [24]. In this review, we focus on biophysical applications of proteins remodeling the cell membrane. While the array of coarse-grained tools can be used to probe cell-biology problems *in silico*, there is also much to be gained from studying these models as a whole, by studying the communication of information between different length scales in biological processes.

## 2. Methods for Parameterizing Coarse-Grained Force Fields

The defining feature of a coarse-grained biophysical model is the length-scale at which chemical components are modeled; such a model necessarily lumps many atomic degrees of freedom into a single coarse-grained bead. As with any classical molecular dynamics (MD) approach, a CGMD model treats molecules classically, integrating Newton’s laws of motion according to potentials, which define the forces between each bead in the system.

(1) $$m_{i}\frac{\partial^{2}r_{i}}{\partial t^{2}}=F_{i},\;F_{i}=-\frac{\partial V}{\partial r_{i}},\;i=1\ldots N$$

These equations describe the motion of *N* particles, each with mass, *m_i_*, experiencing a force, *F_i_*, due to a potential energy function, *V* , itself a function of the configuration of all atoms in the system that are close enough to exert a measurable force. Several software packages are capable of integrating these equations, including the popular GROMACS [25], NAMD [26], CHARMM [15] and AMBER [27] packages. Many of the coarse-grained methods utilize one of these integrators to perform simulations.

Molecular dynamics simulations make contact with observables, like temperature and pressure, via statistical mechanics. Temperature is defined by the kinetic energy of the particles, while macroscopic pressure is defined by the average of the molecular virial [28] as follows:

(2) $$\frac{1}{2}N_{df}k_{B}T=E_{\mathrm{kin}},\;E_{\mathrm{kin}}=\frac{1}{2}\sum_{i}^{N}m_{i}v_{i}\cdot v_{i}$$

(3) $$P=\frac{2}{V}[E_{\mathrm{kin}}-\Xi ],\;\Xi =-\frac{1}{2}\sum_{i<j}r_{ij}\cdot F_{ij}$$

In this equation, 𝒱 is the volume of the system, *E_kin_* is the kinetic energy, **r***_ij_* is the distance vector between particles, *i* and *j*, **F***_ij_* is the corresponding force, *N_df_* is the number of degrees of freedom (3*N* 3 for *N* particles, minus any constraints) and Ξ is the virial. The choice of these forces and the physical quantities they represent—dispersion forces, electrostatics and bonded forces—define the model and determine its ability to reproduce observed physical phenomena. In this section, we will first summarize the early advances in coarse-graining and then review three representative coarse-grained models built from structure-based, force-based and energy-based force-fields, respectively. Because coarse-graining requires a simplification of many degrees of freedom, it is impossible to build a model that simultaneously reproduces the all of the geometric, thermodynamic and kinetics features of a physical system. To build a coarse-grained model, it is therefore necessary to choose which physical properties are essential to the behavior of the target system. We can classify the most popular models by which property they aim to reproduce, namely, the geometry of the system (structure-based), the distribution of forces between particles or thermodynamic properties (energy-based). The representative models described in Sections 2.2 through 2.4 each take these approaches, though there is significant overlap, and these are not the only suitable coarse-grained methods. In fact, much of the power of the coarse-graining method lies in its flexibility and the ease with which it can be adapted to new applications.

### 2.1. Early Coarse-Grained Models and Dissipative Particle Dynamics

The development of coarse-grained models for interfacial systems was made possible by the need to bridge detailed atomistic simulations with continuum methods. The seminal coarse-grained modeling approaches drew from many different methods, including both Monte Carlo and molecular dynamics integration schemes, lattice and off-lattice models and hard sphere, Lennard-Jones potentials. While a comprehensive summary of these modeling approaches is beyond the scope of this review, early development of these models and connections to earlier work is summarized in a pair of reviews [29,30]. In general, early coarse-grained models can be classified by the number of molecules that are mapped onto a single coarse-grained particle.

Early models mapped a single molecule onto one coarse-grained particle in order to simulate spontaneous phase separation. Larson employed a Monte Carlo scheme [31] to simulate oil-water-amphiphile systems on two- and three-dimensional cubic lattices, while Smit and coworkers used molecular dynamics to simulate these systems using Lennard-Jones particles [32]. In 1998, Goetz and Lipowsky modeled surfactant molecules by Lennard-Jones spheres connected by harmonic bonds in order to simulate the self-assembly of bilayers and micelles and calculate the resulting stress and density profiles [33]. With a molecular representation, they calculated the bending rigidity of the bilayer from its fluctuation spectra and demonstrated that these models are able to reproduce both bending and protrusion modes [34].

To reach larger time and length scales, the dissipative particle dynamics (DPD) method uses a much coarser mapping, in which one site may represent many molecules in a small fluid volume [35,36]. There are three types of forces present in DPD models: a conserved soft repulsion force, pairwise dissipation forces and pairwise random forces. The balance of dissipation and random forces provides the thermostat for the DPD model, and since this thermostat preserves the momentum of individual particles, these models provide correct hydrodynamic behavior. In addition to using a coarser mapping, DPD simulations use a longer time-step, due to the use of soft repulsion forces. It is necessary to match the observed compressibility in a DPD simulation to the target fluid in order to study the phase behavior and interfacial tension of the model fluid [37]. The DPD method has been applied to biological lipid bilayers [38], membrane fusion processes [12,39] and bilayers with proteins [40], and its connections to the mesoscale have been reviewed extensively [41–43].

It is clear that the full spectrum of coarse-grained modeling approaches contains contributions from several different fields. Early coarse-grained simulations were made possible by advances in computer hardware, which made it possible to simulate larger interfacial systems at finer levels of detail. These simulations began to bridge the gap between the atomistic simulation of lipids and protein systems with mesoscale, statistical mechanics models for membranes. In this section, we have cited some of the milestones in coarse-grained model development. To review more recent coarse-grained simulation methods and to limit the scope of this review, we will now turn our attention to three models that represent the structure, force and energy-matching approaches. This list is by no means comprehensive, and there are many other coarse-grained models for simulating biological, interfacial systems available in the literature.

### 2.2. Structure and Energy Matching in the CMM-CG Model

In the early 2000s, Klein and coworkers developed a coarse-grained model for phospholipid bilayers by matching the structural and thermodynamic properties of water, hydrocarbons and lipid amphiphile to experimental measurements and all-atom simulations. The resulting force field, titled CMM-CG (“Center for Molecular Modeling Coarse-Grained”) , has been used to investigate a range of polymer systems, as well as those containing nonionic liquids and lipids.

The model was originally developed to reproduce structural properties of a dimyristoylphosphatidylcholine (DMPC) bilayer [44]. This requires careful assembly of water, hydrocarbon and amphiphilic components. Given that water is the largest constituent of many soft-matter systems and, indeed, makes most condensed matter systems truly “soft”, it is necessary to reproduce both its structure and phase transitions. The CMM-CG model maps three water molecules onto a single bead.

Non-bonded forces are modeled with general Lennard-Jones (LJ) potentials with a potential well depth (*ε_αβ_*) and zero-position (*σ_αβ_*), which is tuned to reproduce the desired structure and thermodynamic properties of the target system. The softer 12-4 potential was used to model dispersion forces in water by matching the melting temperature, density and vapor pressure observed in bulk and thin-film test simulations.

(4) $$V(r_{ij})=\frac{3\sqrt{3}}{2}\varepsilon_{\alpha \beta }\;\left\{{\left(\frac{\sigma_{\alpha \beta }}{r_{ij}}\right)}^{9}-{\left(\frac{\sigma_{\alpha \beta }}{r_{ij}}\right)}^{6}\right\}\;(\text{non-bonded})$$

(5) $$V(r_{ij})=\frac{27}{4}\varepsilon_{\alpha \beta }\;\left\{{\left(\frac{\sigma_{\alpha \beta }}{r_{ij}}\right)}^{12}-{\left(\frac{\sigma_{\alpha \beta }}{r_{ij}}\right)}^{4}\right\}\;(\text{water})$$

In the CMM-CG model, the well depth in water-water interactions was chosen to simultaneously provide a melting temperature of 212.1 K, a boiling temperature of 373.15K and to recover the correct density of water at 303.15 K in a three-water bead model [44].

To model soft-matter systems, it is necessary to include hydrophobic chemical components; in this case, represented by a collection of *n-*alkanes. Simulations of alkanes in the CHARMM force field [45] provide target structural data for harmonic bond and angle potentials given by Equation (6). This harmonic bond and angle are standard among many molecular dynamics force fields; a comprehensive summary of many common potential functions can be found in the GROMACS manual [25,46].

(6) $$V_{\mathrm{bond}}(r)=\frac{k_{\mathrm{bond}}}{2}{\left(r-r_{eq}\right)}^{2},\;V_{\mathrm{bend}}(\theta )=\frac{k_{\mathrm{bend}}}{2}{\left(\theta -\theta_{eq}\right)}^{2}$$

In Equation (6), *V_bond_* and *V_bend_* represent the contribution of the bond stretching and bending to the potential energy function, *k_bond_* and *k_bend_* are the corresponding length and angle stiffnesses and *r_eq_* and *θ_eq_* are the equilibrium bond length and angle, respectively. Tuning the equilibrium values and spring constants specify the structure and fluctuations of individual molecules. Likewise, the inter-molecular structure and thermodynamics of the target fluid depends on non-bonded interactions, which are modeled with a 9-6 LJ potential. Nonbonded parameters were chosen to reproduce phase separation with water, as well as experimental bulk density and vapor pressure measurements.

Finally, to assemble hydrophobic, hydrophilic and water components into a viable model for amphiphiles, non-bonded parameters must be chosen to reproduce the physics of lipid bilayers. Classic coarse-grained methods propose pair potentials between coarse-grained (CG) beads, according to the Boltzmann inversion method. In this method, a pair correlation function, or a radial distribution function (RDF), *g*(*r*), defines the probability of finding a particle at distance, *r*, from a reference particle, such that the conditional probability of finding the particle is *ρ*(*r*) = *ρg*(*r*), where *ρ* is the average number density of the fluid. This pair correlation function may be calculated by analyzing an atomistic trajectory mapped onto coarse-grained beads. A potential of mean force (PMF) between CG beads is then estimated by Equation (7), where *g_aa_*(*r*) is the RDF measured from atomistic simulation, *k_B_* is the Boltzmann constant, *T* is absolute temperature and *α_n_* is a scaling factor (corresponding to the *n*^th^ iteration of the estimate) designed to include the effect of interactions with the (necessarily) heterogeneous environment.

(7) $$V_{n}(r)=\alpha_{n}\;\{-k_{B}T\ln(g_{aa}(r))\}$$

The Boltzmann inversion method may be iteratively corrected according to Equation (8) to correct the tabulated potentials until the pair-correlation functions for the atomistic and coarse-grained systems agree.

(8) $$V_{n+1}(r)=V_{n}(r)+k_{B}T\ln\frac{g_{n}(r)}{g_{aa(r)}}$$

In practice, since the pair correlation function computed in an homogeneous environment is not equivalent to the potential of mean force in an inhomogeneous environment, it is necessary to include the effects of correlated contributions from the surrounding environment, as well as contributions from bonded intra-molecular forces. To account for these contributions, a reverse Monte Carlo (MC) method proposed by Lyubartsev and Laaksonen [47] is used to construct an effective pair potential. In the canonical ensemble, we may write the expectation for an observable RDF as:

(9) $$\langle g_{n}(r_{i})\rangle =\frac{\int g_{n}(r_{i})e^{-\beta V}dr}{\int e^{-\beta V}dr}$$

where *β* is the inverse temperature, (*k_B_T*)^−1^. In the following formulation, *j* indexes atoms or sites, the *r_i_* refer to a set of inter-site distances, which define the RDF, and *n* indexes the iterated calculations of the RDF given by *g_n_*(*r_i_*). Taking the partial derivative gives the following fluctuation formula.

(10) $$\frac{\partial \;\langle g_{n}(r_{i})\rangle }{\partial V(r_{j})}=-\beta \;[\langle g_{n}(r_{i})g_{n}(r_{j})\rangle -\langle g_{n}(r_{i})\rangle \;\langle g_{n}(r_{j})\rangle ]$$

This relates changes in the coarse-grained RDF to changes in tabulated potentials, while considering cross-correlations between subject particles and their environment. Linearizing this equation gives a solution according to Equation (11), which will yield self-consistent pair potentials in agreement with the atomistic data.

(11) $$\langle g_{n}(r_{i})\rangle -g_{\text{target}}(r_{i})=\sum_{j=1}^{N}\frac{\partial \;\langle g_{n}(r_{i})\rangle }{\partial V_{n}(r_{j})}\Delta V_{n}(r_{j})$$

It can be shown that iteratively solving for these potentials, *V_n_*, with Monte Carlo methods (known as reverse Monte Carlo) can generate effective pair potentials, which reproduce soft matter properties for a particular system. However, this method suffers from reduced transferability, because it depends on a particular thermodynamic ensemble. That is, the target RDF includes information about temperature, density and, most importantly, composition, which limits its applicability to novel systems. For this reason, it is necessary to test the model against thermodynamic data.

The relevant thermodynamic property in amphiphilic systems is the surface tension, which can be calculated from MD simulation by Equation (12), where *L_z_* is the box-size normal to the interface and *P_ij_* is the *ij* component of the pressure tensor. Its condensed phase analog is the interfacial tension, which can be estimated with a combination of experiment and theory [48]; however, these measurements are subject to large errors.

(12) $$\gamma =\frac{L_{z}}{2}\langle P_{zz}-\frac{P_{xx}+P_{yy}}{2}\rangle$$

Matching the area-per-lipid and bilayer density (or electron density profile if matching to AAMDsimulations or neutron diffraction) confirms that the CGMD has the correct structure. Tuning non-bonded cross-terms between lipid headgroups and hydrophilic tails will influence the observed surface tension or its conjugate variable, area-per-lipid, in simulations under zero tension. Capturing both condensed phase structure and energies in this way is necessary for building an accurate model. Contact with experiments will be discussed further in Section 2.5.

### 2.3. Force Matching with the Multiscale Coarse Grained (MS-CG) Model

Gregory Voth and coworkers have proposed the concept of force-matching to develop a rigorous coarse-grained force field directly from forces measured in all-atom simulations. This is necessary, they argue, because other coarse-grained approaches suffer from reduced transferability compared to all-atom counterparts, namely because the coarse-grained simulation may not contain the correct thermodynamic ensemble. Insofar as the multi-body coarse-grained PMF is derived from structure factors that depend on temperature, pressure and composition, they cannot be transferred to new systems.

To avoid this problem, they propose a variational method in which a coarse-grained force field is systematically developed from all-atom simulations under the correct thermodynamic ensemble [49]. In the statistical framework developed by Izvekov and Voth [49–51], it is possible to develop the exact many-body coarse-grained PMF from a trajectory of atomistic forces with a sufficiently detailed basis function.

In the original force-matching method developed by Ercolessi and Adams [52], a set of parameters defining classical forces of a pre-defined form are optimized by minimizing their squared difference from reference forces provided by *ab initio* simulation. This fitting procedure becomes intractable with the many components found in biochemical systems. To circumvent this optimization problem, Voth *et al.* have designed the force field to be linear in the fitting parameters by constraining their choices of basic functions to those that have zero derivatives between mesh points. This allows one to optimize the force field by finding the least-squares solution to an overdetermined system of linear equations.

They start with a collection of sampled configurations from an atomistic simulation of the target system and calculate the reference forces between atoms of a particular type. After decomposing their target force into a short-ranged part approximated by a cubic spline and a long-ranged Coulomb part, they solve the overdetermined set of linear equations given by Equation (13).

(13) $$\sum_{\beta =1}^{K}\sum_{j=1}^{N_{\beta }}\left(-f(r_{\alpha il,\beta jl},\{r_{\alpha \beta ,\kappa }\},\{f_{\alpha \beta ,\kappa }\},\{f_{\alpha \beta ,\kappa }^{\prime \prime }\})-\frac{q_{\alpha \beta }}{r_{\alpha il,\beta jl}^{2}}\right)\;n_{\alpha il,\beta jl}=F_{\alpha il}^{\mathrm{ref}}$$

In Equation (13), the {*r_αβ, κ_*} correspond to the spline mesh at points *κ* for pairs of atoms of type *α* and *β;*, while { $f_{\alpha \beta ,\kappa }f_{\alpha \beta ,\kappa }^{\prime \prime }$} are spline parameters that ensure continuous derivatives, *f′*(*r*), at the mesh points and define the short-ranged part of the force. The subscript, *αil*, labels the *i*-th atom of type *α* in the *l*-th sampled atomic configuration. Solving these equations minimizes the Euclidean norm of vectors of residuals and can be solved on a minimal set of atomistic snapshots using a singular value decomposition (SVD) algorithm [53]. By adding the Coulomb term to the short-ranged potential above, this technique allows for the inclusion of explicit electrostatics. The MS-CG model reproduces site-to-site RDFs from atomistic MD simulations, as well as the density profile perpendicular to the bilayer normal in DMPC bilayers [49].

The MS-CG method has been extended to access still larger time and length scales using an approach called hybrid analytic-systematic (HAS) coarse-graining. In this method, the MS-CG force field provides the in-plane center-of-mass lipid interaction potentials, while an analytic Gay-Berne (GB) liquid crystal model describes any inter-monolayer and out-of-plane interactions. The GB liquid crystal model uses ellipsoidal particles, which interact with an anisotropic form of the Lennard-Jones 12-6 potential, and has been successfully applied to higher-resolution coarse-grained modeling with explicit water [54]. In the HAS approach, however, the GB interactions replace those with explicit solvent, providing significant computational efficiency. This model successfully self-assembles and reproduces the undulation spectrum, tensionless area per lipid and area compressibility modulus in agreement with experimental measurements. It has been used to simulate a ~200 nm liposome [55] and N-BARprotein remodeling of a liposome [56].

### 2.4. The Energy-Based Approach of the Martini Force Field

The Martini force field developed by Siewert-Jan Marrink and co-workers eschews systematic structure-matching in pursuit of a maximally transferable force field, which is parameterized in a “top-down” manner, designed to encode information about the free energy of the chemical components, thereby increasing the range of thermodynamic ensembles over which the model is valid. To date, it has been used to study a broad range of biological soft-matter systems described in Sections 2.5, 3.2.2. and 4.

The Martini model employs a four-to-one mapping of water and non-hydrogen atoms onto a single bead, except in ring-like structures, which preserve geometry with a finer mapping. Molecules are built from relatively few bead types, which are categorized by polarity (polar, non-polar, apolar and charged). Each type is further distinguished by hydrogen bonding capabilities (donor, acceptor, both or none), as well as a score describing the level of polarity. Like the CMM-CG and MS-CG models, Lennard-Jones parameters for non-bonded interactions are tuned for each pair of particles. These potentials are shifted to mimic a distance-dependent screening effect and increase computational efficiency. Charged groups interact via a Coulomb potential, $U_{\mathrm{elec}}(r)=\frac{q_{i}q_{j}}{4\pi \varepsilon_{0}\varepsilon_{r}r}$, with a low relative dielectric of (&*espiv;_r_* -= 215) for explicit screening. This allows the use of full charges, while reproducing salt structure factors seen in previous atomistic studies [16], as well as the hydration shell identified by neutron diffraction studies [57]. Non-bonded interactions for all bead types are tuned to semi-quantitatively match basic measurements of density and compressibility [58].

Bonded interactions are specified by potential energy functions, which model bonds, angles, dihedrals and impropers with harmonic functions, with relatively weak force constants to match flexibility of target molecules at the fine-grained resolutions.

(14) $$V_{b}=\frac{1}{2}K_{b}{\left(d_{ij}-d_{b}\right)}^{2}\;(\text{bond})$$

(15) $$V_{a}=\frac{1}{2}K_{a}{\left(\cos(\phi_{\mathrm{ijk}})-\cos(\phi_{a})\right)}^{2}\;(\text{angle})$$

(16) $$V_{d}=K_{d}{\left(1+\cos(\theta_{\mathrm{ijkl}}-\theta_{d})\right)}^{2}\;(\text{dihedral})$$

(17) $$V_{id}=K_{id}{\left(\theta_{\mathrm{ijkl}}-\theta_{id}\right)}^{2}\;(\text{improper}\;\text{dihedral})$$

Here, *V* represents the component of the potential energy function arising from bond, angle, dihedral and improper dihedral contributions, the set, *fK_b_; K_a_; K_d_; K_id_g*, represents the corresponding stiffness constants, and the set, {*d_b_; ϕ_a_; θ_d_; θ_id_*}, represents the equilibrium values for these interactions. Dihedralpotentials are only implemented for peptide backbones. Alkanes are constructed to reproduce and bond and angle parameters given by atomistic simulations in the GROMOS force field [59].

Simulations of small ice cubes surrounded by water show that Martini ice is in equilibrium with liquid water at 290 K and melts within 5 K of this temperature. Like many CG models, Martini water becomes supercooled as the temperature is lowered, failing to freeze spontaneously until 240 K [60].

The defining feature of the Martini force field is the selection of non-bonded parameters, which are optimized to reproduce thermodynamic measurements in the condensed phase. Specifically, the Martini model semi-quantitatively reproduces the free energy of hydration, the free energy of vaporization and the partitioning free energies between water and a collection of organic phases, obtained from the equilibrium densities in both phases: Δ*G^oil=aqueous^* = *k_B_T* ln (*ρ_oil_=ρ_aqueous_*). These calculations require long MD simulations of two-phase systems with very dilute concentrations of the target substance. Results agree to within 2*k_B_T* for many of these properties [16].

Systematic tuning to experimental partitioning free energies was used to select particle types for amino acids, represented with up to four beads, which correspond to the polar character of the amino acid. Additionally, the pre-determined secondary structure modulates the character of the beads; backbone hydrogen bonds found in helices have reduced polar character [16]. While the building blocks for the Martini model were chosen to match thermodynamic data in general, any application of Martini model can be optimized by comparison to AAsimulation, especially when designing bonded interactions to reproduce the protein structure.

In the Martini model, calculation of the partitioning free energy of water in hexadecane agrees with the measurement of 25 kJ mol ^1^ observed in Fischer titration [61]. More broadly, a combination of experiments and predictive modeling efforts have quantified partitioning free energies for others [62,63] and contributed to the parameter choices made by Marrink *et al.* [16]. Spin-echo nuclear magnetic resonance experiments provide self-diffusion data for water and alkanes [64,65].

The hydration free energy can be calculated by comparing the partitioning of target molecules between liquid and vapor water phases, while the vaporization free energy can be calculated from the simulation of liquid-vapor equilibrium. In these simulations, concentrations of 0.01 mole fraction provide a reasonable approximation of infinitely dilute solutions. A comparison of thermodynamic properties by Baron *et al.* [66] showed that this CG model tends to overestimate the water-oil repulsion with free energies of vaporization and hydration, which are systematically high, but still follow the correct trend.

The surface tension measures the free energy cost of adding area to the interface between solvents. Simulations were compared to drop volume tensiometry measurements with good agreement for water, vapor and dodecane mixtures [67]. Electron density profiles from X-ray diffraction data on multilamellar arrays of bilayers provide a measure of the thickness. While neutron scattering is weaker, specific deuteration of different lipid components gives a local contrast agent without chemical modifications [68].

It is clear that the coarse-grained models described in this review often share the same target data. In this section, we have summarized the most important experiments, which inform the Martini model in order to demonstrate the breadth of the physics that these data capture. While the three CG models reviewed in this paper produce extremely rich physics, they draw on data sets with orders of magnitude of greater detail and information. Some of the key differences between these models are summarized in Table 1 . For this reason, there is no one “correct” method for incorporating these data into an accurate coarse-grained model. Indeed, it is impossible for any coarse-grained model to simultaneously match thermodynamic, structural and kinetic features perfectly. Therefore, it is necessary for model developers to choose specific experimental results, which are relevant to the desired application. In the next section, we will take a deeper look at how these experiments can be used in a general coarse-grained model.

### 2.5. Reproducing Experiments in Coarse-Grained Models

The coarse-grained model-development process described in the preceding sections is often iterative. Beginning with first-principles estimates for potential energy functions, these functions are initially parameterized from fundamental structure and thermodynamics measurements. Since the function between these parameters and higher-order experimental observables is unknown by definition, the model is then iteratively tuned to match these experimental target data. In this section, we will summarize the range of experiments that can validate a coarse-grained model.

#### Titration

One of the guiding principles for energy-based coarse-graining is that the model should show the correct partitioning free energies of its constituents, since this property describes the attraction or repulsion between phases. This quantity can be measured by titration methods that determine the density of one species in a fluid of the other, at equilibrium. For example, Karl Fischer titration of water in hydrocarbons [61,70] is the target data for alkane-water interactions in the Martini model. Salt solution diffraction experiments show two hydration shells around these ions, and this justifies the inclusion of hydration-shell waters in coarse-grained ion models [57]. These methods may be extended to more complex systems, namely the partitioning of amphiphilic solutes onto membranes, using titration calorimetry [9,71,72].

#### Magnetic Resonance Spin Echo

The self-diffusion coefficient is the diffusion rate of a particle when its chemical potential gradient is zero, given by $D_{i}^{*}=D_{i}(\partial \ln c_{i}/\partial \ln a_{i})$, where *c_i_* is concentration, *a_i_* is activity and *D_i_* is the diffusion coefficient of the species [73]. Because the diffusion rate reflects the chemical potential, modeling it correctly helps ensure the correct equilibrium density of the fluid. The self-diffusion coefficient for a particle type, *i*, may be measured in simulations via Einstein’s diffusion equation, which relates the diffusion constant to the mean-squared displacement of the particle over time. It is given by the following equation, where *N* is the number of particles, *r_j_*(*t*) is the position of particle, *j*, and time, *t*, and the brackets denote an ensemble average over all starting times.

(18) $$D=\lim_{t\to \infty }\frac{1}{6Nt}\langle \sum_{j=1}^{N}{\left(r_{j}(t)-r_{j}(0)\right)}^{2}\rangle$$

Self-diffusion coefficients can be measured with magnetic resonance spin echo [64,65,70]. In a nuclear magnetic resonance (NMR) spin-echo experiment, the self-diffusion is a proportionality constant, which relates the logarithm of echo intensity and the strength of the magnetic field gradient. These experiments make direct contact with CGMD simulations, which quantify diffusion rates by the slope of the mean-squared displacement. For example, the Martini polarizable water molecule matches experimental diffusion coefficients [74].

#### Neutron and X-ray Scattering

The behavior of salts in water has a strong effect on the electrostatics of any coarse-grained system. Because neutrons are electrically neutral and interact only with the nucleus of a particular atom, they are subject to very short-ranged interactions and, therefore, penetrate the sample very efficiently. In scattering experiments, incident particles impart and receive momentum energy from the sample and scatter at a measurable angle. Measuring the number, angle and energy (the scattering intensity) of the diffracted neutron provides a time- and space-dependent auto-correlation function via Fourier transform. In this way, neutron scattering is capable of measuring the density and time-dependent correlations of a fluid. This resulting dynamical structure factor can be directly compared with the radius of gyration in order to tune non-bonded interactions in simulations. These methods are reviewed by Fischer *et al.* [75]. Neutron scattering can provide a density profile of the bilayer; however, only recent experimental methods have measured this density in fully hydrated bilayers [76].

X-rays may be used in place of neutrons in order to measure the electron density profiles of lamellae in vesicles and stacks. Small-angle X-ray scattering (SAXS) is used to quantify molecule size, low-angle X-ray scattering (LAXS) provides electron density profiles and wide-angle X-ray scattering (WAXS) can measure in-plane features of the bilayer, including micro-domains [68,77]. Both neutron and X-ray scattering can provide density profiles, which describe the thickness of a bilayer. Combined with volume information, these can be used to estimate the area-per-lipid, which is a crucial structural feature of a bilayer simulation. The range of scattering experiments is often difficult to match to biologically relevant systems, and different methods often produce conflicting results. Specific algorithms for fitting these data to CGMD simulations are under development [78].

Significant effort must be devoted to faithfully reproducing the area-per-lipid in bilayer simulations not only to match experimental structure measurements, but because interfacial area and surface tension are conjugate thermodynamic variables. Recent re-parameterization of the CHARMM force field for lipids [15] optimizes the lipid partial charges and Lennard-Jones parameters using QMcalculations and experimental data to optimize the area per lipid in the tensionless ensemble. A survey of integrator parameters in GROMACS for a number of force fields [79] also provides guidance for choosing correct short- and long-range cutoffs and water models necessary to achieve the correct lipid areas and tensions in the correct phase.

#### Nuclear Magnetic Resonance

In order to characterize the order in lipid hydrophobic tails, one can measure the angle between a chemical bond and the bilayer normal. This gives the second-rank order parameter, $P_{2}=\frac{1}{2}(3\cos^{2}\theta -1)$. Deuterium magnetic resonance (DMR) experiments use selective deuteration of carbon atoms in the hydrophobic tails. The order parameter is a function of the residual quadropole coupling value. DMR does not require sonication, in contrast with spin-label NMR measurements [80]. Coarse-grained lipids models do not include all of the available order-parameter data, because they possess fewer degrees of freedom in their tails; instead, angles measured in CGMD tails may be tested for agreement with atomistic simulations, which must reproduce the order parameter.

#### Phase Transition and Tension Measurements

The area per lipid and surface tension are conjugate variables; all other things being equal, fixing one should fix the other, for a particular thermodynamic ensemble. Given that these properties depend strongly on the chemical composition (lipid type, hydration level) of a particular system, reproducing the phase transition temperatures is a useful indicator that the model is robust.

Most phase transition data is provided by measuring density via scattering or order parameters via NMR at various temperatures. To observe the phases directly, epifluorescence microscopy improves upon these measurements by making it possible to resolve microdomains and phase coexistence in monolayers [81]. Cryo-transmission electron microscopy (cryo-TEM) and differential scanning calorimetry are also used to characterize the phase behavior of bilayers [82]. Densitometry, and acoustic measurements have been used to study dipalmitoylphosphatidylcholine (DPPC) multilamellar vesicles, extracting the temperature-dependence of phase transitions and plausible theories for the kinetics of these transitions [83]. This topic is described further in Section 4.1.

In monolayer systems, the pressure-area isotherm can be measured via a Langmuir trough or captive bubble surfactometer. These measurements can be mimicked in coarse-grained simulations in order to validate the model against experiments, quantify finite size effects and investigate the effects of composition on the isotherm [84] and the dynamics of monolayer buckling [69,85]. Since bilayers are unstable when lipids include fewer than nine carbon atoms per tail, the observation of spontaneous pore formation in the Martini model indicates that the balance of hydrophobic repulsion and entropic repulsion qualitatively matches experiments. This balance is also confirmed via calculation of the lateral stress profile, which is compared to atomistic simulation [16].

More generally, calculations of line tension in bilayers with mixed compositions agree semi-quantitatively with those measured by fitting fluorescence microscopy of mixed composition giant unilamellar vesicles (GUVs) to elastic theory or micro-pipette aspiration [86,87]. Another measure of bilayer stability is the water permeation rate. Permeability coefficients from vesicles can be measured via micro-turbimetry and differential scanning calorimetry. These rates also help set the time-scale of the coarse-grained simulation [60].

Simulations have the ability to measure local pressure from the first moment of the stress profile [88]. When averaged across the bilayer plane, this gives the stress profile, which may be compared to atomistic simulations. The integral of the stress profile along the bilayer normal, *z*, between *z*_1_ and *z*_2_ across the mid-plane, *z*_0_, also quantifies the intrinsic curvature, *C*_0_, via $\kappa C_{0}=\int_{z_{1}}^{z_{2}}(z-z_{0})\sum (z)dz$. In this formulation, the local pressure tensor, **P**(*r*), gives the lateral pressure profile by $\sum (z)=\frac{1}{2}(P_{xx}+P_{yy})-P_{zz}$. These calculations have made it possible to tune the balance of forces in Martini model bilayers in qualitative agreement with atomistic simulation [16]. Given an estimate for bending rigidity, the resulting spontaneous curvature values agree with the fluorescence spectroscopy of supported lipid bilayers experiments for several lipid types [89]. Calculation of the local stress tensor also makes it possible to study the position-dependent stress profile generated by membrane-protein systems, such as the mechano-sensitive protein channel, MscL [3,90]. Applying a similar method to the study of the stress profile in atomistic simulations makes it possible to quantify both the chemical and entropic contributions to the tension [91].

The integral of the lateral stress profile itself gives the surface tension ( $\sigma =-\int_{z_{1}}^{z_{2}}\sum (z)dz$) of the bilayer. Because the tension and its conjugate variable area-per-lipid determine the phase of the system, there has been much debate on choosing the correct ensemble for biological simulations of lipid bilayers. In experiments, bilayers adjust their area per lipid to minimize contact between water and hydrophobic lipid tails. For bilayers with no spontaneous curvature, this gives a free energy minimum, (*∂G*/*∂A*)*A*_0_ = *γ*, which must be zero. Some have argued that coupling between area and thickness may introduce another variable, changing the free energy dependence to include non-zero tension. However, in practice, this coupling is very weak, and simulation studies find good agreement with experiments when using zero tension [92–95].

#### Measuring Elastic Properties

Lipid bilayers possess an incredible combination of material properties that make them ideally suited to hosting biophysical processes and compartmentalizing the cell. Several experimental methods are able to measure the elastic properties of a bilayer. Many of these methods use the Helfrich model to interpret their results [96]. In this model, the membrane is treated as an infinitesimally thin elastic sheet with energy terms from bending, Gaussian curvature and surface tension. It is given by Equation (19), where *κ* is the bending modulus, *H* is the mean curvature, *H*_0_ is the intrinsic curvature (zero for a symmetric bilayer), *G* is the Gaussian curvature, *κ̄_G_* is the Gaussian bending modulus and *γ* is surface tension.

(19) $$H_{\text{el}}=\int \left\{\frac{\kappa }{2}{\left(H-H_{0}\right)}^{2}+{\bar{\kappa }}_{G}G+\gamma \right\}\;dA$$

The Helfrich approximation forms the basis of many mesoscale models, briefly described in Section 22 . The bending rigidity, *κ*, describes the energy required to bend the membrane to a unit curvature and is usually estimated at ~ 20*k_B_T* for biological lipid bilayers. Because the Gaussian curvature is invariant under deformations, it is relevant only to topology changes in the membrane, *i.e*., vesicle fusion or phase transitions, or when the Gaussian rigidity varies along the spatial coordinate. The bending modulus (or bending rigidity) can be measured in a number of ways. The Fourier transform of the Helfrich Hamiltonian given by Equation (20) measures the height-height auto-correlation function (otherwise known as the undulation spectrum). For bilayers with nearly zero surface tension (~0.1 N/m), it is possible to fit this function in the low-*q* regime, where *κ* may be extracted as a pre-factor.

(20) $$\langle |u_{\text{und}}(q){|}^{2}\rangle =\frac{k_{B}T}{A(\kappa q^{4}+\gamma q^{2})}$$

Here, *q* is the transformed variable defining the Fourier transform, and $u_{\text{und}}^{2}(q)$ is the height-height auto-correlation function. These fluctuations can be directly calculated from video phase contrast microscopy, which quantifies the bilayer shape changes according to the fluctuation spectra given by Equation (20) [97]. Shear flow experiments on giant vesicles can be used to measure the bending rigidity by relating the deformation of the vesicle to the flow in a theory that includes thermal membrane undulations [98]. Micropipette aspiration experiments provide a measure for bending rigidity, as well as the area compressibility modulus, which is given by $K_{A}=A_{0}^{*}{\left(\partial \gamma /\partial A_{0}\right)}_{T}$, where *A*_0_ is the area per lipid and $A_{0}^{*}$ is the area per lipid at the free energy minimum. This study indicates that bending rigidity increases with the number of carbons [99]. In addition to the bending modes, contributions to the energy arise from peristaltic modes corresponding to fluctuations in the inter-leaflet distances and protrusion modes corresponding to lipid motion normal to the bilayer plane (and, therefore, high wave-number modes in Equation (20)). In most cases, these modes are decoupled from the bending modes. Due to the high resolution provided by bilayer simulations, recent studies have characterized these undulations, peristaltic motions and area compressibilities for comparison with experiments and a better understanding of membrane elasticity [93,94,100–102].

Cholesterol molecules, present in biological membranes, induce changes in bilayer elasticity and a reduction in the area per headgroup. This effect has been investigated using a number of atomistic force fields and can be reproduced in coarse-grained models [16]. Experiments that use fluorescent quenching methods can be used to investigate the phase coexistence in bilayers with cholesterol [103,104], providing useful target data for cholesterol coarse-graining.

#### Summary

In this section, we have reviewed many of the experiments that inform coarse-grained models for soft-matter systems; these methods are briefly summarized in Table 2. It is important to note that coarse-grained models can be designed to reproduce many other experimental methods beyond the scope of this review. Soft-matter experiments are rich in information about the structure and dynamics of the components of many biological systems; however, it is far from easy to design models that reproduce these quantities. Even when the experimental data are clear, there is no guarantee that a coarse-grained model will be able to capture the nuance and context of these experiments without careful adjustment and attention to the limits of the model. The limits of coarse-grained simulations of soft matter will be discussed in the next section.

### 2.6. Assessing CGMD Model Performance

In the preceding sections, we have described the construction and verification of representative coarse-grained models. Comparison to basic experiments verifies that the model is capable of matching basic physical properties of soft matter systems. In the remainder of this review, we will discuss the validation of these models on more complex biomolecular systems. Any coarse-grained model is only useful insofar as it can reproduce the physics of a complex system. In this sense, the model must be carefully designed to match experiments and, more importantly, answer a clear question about a biophysical system. In this section, we will note some of the limitations inherent to the coarse-grained modeling approach.

The clearest limitations of a coarse-grained model are the result of discarded degrees of freedom. For example, the earliest iterations of the CMM-CG model lacked explicit electrostatic interactions. The standard (non-polarizable) Martini model includes electrostatics; however, they are highly screened and, thus, imprecise compared to all-atom methods. In the residue-based coarse-graining approach used to model membrane remodeling by N-BAR (see Section 4.2 ), an artificially low dielectric constant was necessary to reproduce the electrostatic interactions between the protein and bilayer [2,105,106].

It has been noted that the Martini method is a free energy method and, therefore, includes significant entropy loss owing to the loss of degrees of freedom in the coarse-grained mapping. Enthalpy terms compensate for this; however, the entropy/enthalpy balance may be upset, affecting temperature dependence. Secondary structure is also static in this model; however, many applications can show the relative movement of secondary structure elements. For example, the tension-driven activation of a mechanosensitive channel can be resolved by CGMD [3,107]. Both the inability to model protein conformational change and the challenge of reproducing bilayer physics without an accurate entropy-enthalpy balance are both consequences of the loss of detail in a CGMD model and are therefore common to all of the methods discussed in this review. Similarly, a coarse representation of amino acids often obscures the chemical detail responsible for protein function. We will describe CGMD models for a number of protein-mediated processes in Section 4.

A major objective of coarse-grained modeling approaches is to design transferable or universal force fields capable of modeling novel systems with minimal modification. All-atom force fields do not require extensive tuning, partly because they already contain explicit degrees of freedom for first principles-based interactions. However, not all degrees of freedom are created equal, and a major challenge of coarse-graining is deciding which degrees of freedom are essential to the system of interest. The force-matching (MS-CG) approach addresses this limitation by computing a coarse-grained force field directly from an atomistic trajectory of the target system. A recent survey of MS-CG peptide models indicates that transferability between systems is strongest in the low-energy regions of the free energy landscape [22]. The contrasting bottom-up and and top-down coarse-graining methods highlight the tension between including unnecessary detail and designing universal models. As with any modeling strategy, the choice of a highly-tuned, yet non-transferable model *versus* a general, transferable model will depend on the system of interest.

Coarse-grained molecular dynamics also often fail to reproduce both correct or even self-consistent kinetics. Martini simulations use a time step of 20–40 fs, which is effectively three- or four-fold longer in real time due to the smoothed interactions between CG beads. This speed-up factor may change with system composition. Since CGMD force fields may independently reproduce either the potential energy or free energy of different parts of the underlying system, they may produce incompatible kinetics within the same system. By collapsing many degrees of freedom into relatively few, this model makes a number of compromises. Lack of atomistic detail prevents strong hydrogen bond networks from forming, and this, in turn, generates lateral diffusion rates, which are much higher than normal. For that reason, the time scale of a CG simulation must be calibrated to the diffusion in an AAMD simulation to *a posteriori* determine the duration of the simulation. As a result of model approximations, the relative kinetics for subsets of particles in the same CGMD simulations may not be preserved. Loss of detail also prevents accurate conformational sampling of proteins and accurate reproduction of lipid order factors. Many of these disadvantages may be mitigated by comparison to atomistic systems, experiments and even mesoscale continuum models. Furthermore, despite these limitations, coarse-grained models are able to predict and describe an amazing array of biomolecular systems. Before we discuss these applications, we must first explain the ways in which protein detail can be added to the system.

## 3. Modeling Proteins

The challenges in reproducing the physics of soft-matter systems with a coarse-grained force-field are significantly larger when introducing proteins. In addition to introducing a multiplicity of additional chemical interactions with lipids, it is a significant challenge to successfully model the internal structure and dynamics of membrane proteins. All-atom molecular dynamics simulations provide the best means of capturing these motions, and recent years have seen sophisticated methods for matching these simulations to experiments, NMR in particular. Thanks in large part to the study of all-atom MD simulations of proteins, coarse-grained force fields have incorporated parameters for amino acids. The resulting CGMD applications rely on the wide body of atomistic protein simulation. However, comparing the results of simulations at atomistic and coarse resolutions tells us precisely which kinds of chemistry and nanoscale physics manifests itself at much larger length scales, and how this information is propagated. This information can often be used to guide the design of more useful atomistic simulations.

### 3.1. Atomistic Simulations of Proteins

The earliest simulations of proteins in atomistic detail employed first principles to study proteins *in vacuo* and in solution. Since the first simulation of a protein in 1976 [1], protein simulations have characterized the conformations of proteins, DNA and other biomolecules. A comprehensive review of atomistic protein simulations is beyond the scope of this review; however, summaries of significant progress in the field have been compiled in a series of recent reviews [108–115]. The CHARMM, GROMOS, AMBER and OPLS-AA protein force fields are each capable of simulating biomolecules with similar, but not exact, results. Recent studies have systematically compared these force fields to each other and relevant experiments in order to validate both the molecular dynamics method, in general, and the force field tools, specifically [79,116–120]. The methods for designing coarse-grained force fields described in Section 2 rely heavily upon these force fields.

Atomistic simulations are more “literal” than CGMD simulations, in that they seek to reproduce a particular thermodynamic ensemble with no ambiguity (beyond that of the reference state) in the definitions of physical quantities, such as length, time, force and energy. To this end, they employ first principles often gleaned from quantum mechanical theory and experiment, namely electrostatic potentials, dipole moments and dimerization energies. Atomistic protein simulations also seek to reproduce a host of target data, also used in CGMD parameterization, including spectroscopy data, thermodynamic data, such as solvation free energy and heats of vaporization and X-ray, electron and microwave diffraction structures. With meticulous matching to experiment and quantum mechanical theory, and consequent application in parameterization of CGMD force fields, atomistic simulations provide a filter through which these experimental data inform CGMD models.

Having matured in recent years, atomistic simulations are now capable of accurately modeling protein folding [18,121–124], predicting protein-ligand docking to guide drug design [112,125–131] and understanding protein mechanical properties [132,133]. Notably, simulations have recently been used to study enzyme binding processes [134], cooperative protein folding [135], the molecular motors [136], solvent behavior in the ribosome exit tunnel [137] and protein-DNA binding [138,139].

#### 3.1.1. Enhanced Sampling Methods

Despite these successes, atomistic molecular dynamics simulations are tempered by the primary disadvantage of atomistic protein simulation: accessing physically relevant time scales. While much work has been devoted to parallelization algorithms, use of graphical processing units (GPUs) and the development of larger, more specialized and massively distributed supercomputers [18], these efforts are unlikely to provide access to large biomolecular systems for more than milliseconds of real time.

The most straightforward way to improve the sampling of a molecular dynamics simulation is to simulate multiple copies of the same system using slightly different starting configurations. This provides a more robust sample, albeit at the same cost as the original simulation. To efficiently extend the atomistic methodology to longer time scales, a collection of enhanced sampling methods has been developed. For example, graph-based geometric methods, probabilistic road maps and Markov models may be used to better understand protein dynamics and kinematics by discarding uncorrelated, high-frequency atomic motions [111]. Elastic network models and normal mode analysis methods reveal collective motion and allosteric mechanisms in good agreement with NMR and X-ray scattering data [113] and often in conjunction with standard coarse-graining [140–142]. Methods, such as transition path sampling, transition interface sampling, forward flux sampling and weighted ensembles, provide additional access to longer time scales in atomistic systems [143]. In replica exchange molecular dynamics, multiple weakly-coupled simulations of the same system are exchanged between temperatures to escape kinetic traps [144]. Biasing potentials may be used to generate non-Boltzmann-distributed ensembles from which equilibrium properties may be calculated in steered molecular dynamics [145]. Metadynamics [146], temperature-accelerated molecular dynamics [147] and other free energy perturbation methods [131,148] and even Monte Carlo methods [149–151]. Perhaps the most thorough extension of atomistic molecular dynamics is realized by coupling simulation with NMR measurements, which enhance the sampling of the simulation at the longer time-scales possible in experiments [152,153].

While these methods seek to extend the range of a particular simulation method, it is also possible to optimize existing coarse-grained approaches using a relative entropy-based method [154]. Many of these enhanced sampling methods discussed in this section may also be used in combination to tackle specific biological modeling problems. Common to each is a reduction in the number of degrees of freedom, making it possible to simulate large atomistic systems in great detail. In that sense, coarse-grained molecular dynamics simulations are another enhanced sampling method, in this case, ideally suited to extending the size and duration of protein simulations.

#### 3.1.2. Atomistic Simulations of Membrane Proteins

Atomistic simulations of membrane proteins provide a direct link between coarse-grained descriptions of large soft-matter systems and the high levels of detail available from atomistic simulations. Reviews of membrane-protein simulations include descriptions of both fine and coarse resolutions, often describing the ways in which these simulations can be linked [155,156].

The study of G-protein-coupled receptors (GPCR) provides the prototype for atomistic investigation, because the action of these transmembrane proteins is modulated by membrane environment. A recent review of atomistic GPCR simulations [157] emphasizes the importance of developing accurate models for lipids and protein oligomerization in order to produce models that can inform experiments and future drug design. In a similar application, circular dichroism experiments at high temperature showed that some transmembrane peptides are thermostable, allowing elevated temperature simulations, which quantified the pathway by which these proteins partition into membranes [72].

Due to the added difficulty of accessing biologically relevant time scales for both proteins and bilayers in combined systems, atomistic membrane protein simulations are often combined with other methods. Atomistic simulation augmented with Monte Carlo methods was used to accelerate lipid equilibration to investigate hydrophobic mismatch near helical peptides [158]. Amphipathic polymers, which stabilize membrane proteins in solution, employed combined all-atom and coarse-grained resolution with a back-mapping scheme to probe particle assembly, in agreement with small-angle neutron scattering [159]. In a study of phospholipase, coarse-grained simulations served as seeds for atomistic simulations, which improved conformational sampling of the peptide [160]. Other studies used atomistic simulation to test lipoprotein complexes against SAXS data [6]. For bilayer systems, in general, reverse coarse-graining makes it possible to connect CGMD models to low angle X-ray scattering (LAXS) measurements of bilayer geometry by confirming these geometries in atomistic simulations [78]. Likewise, the back-mapping from simulations of the antimicrobial peptide alamethicin from the Martini model to the CHARMM27 all-atom force field helped confirm that this peptide loses its helical character during aggregation, in agreement with NMR measurements [161].

The abundance of atomistic membrane-protein simulation reflects the usefulness of high-resolution simulation of membrane-associated proteins. Coarse-grained simulations of proteins serve to extend these studies to larger systems and biologically relevant time scales.

### 3.2. Parameterization of Coarse-Grained Proteins

Introducing protein detail to a coarse-grained force field requires an accurate model for both the structure and dynamics of the protein itself, as well as the interactions with surrounding lipids and solvent, which remain faithful to experimental observations. In this section we will summarize the development of coarse-grained protein models, the experiments they match and their integration into popular force-fields.

#### 3.2.1. Structure-Based Coarse-Grained Protein Modeling

While coarse-grained simulations have difficulty reproducing secondary structural transformations, it is possible to recover accurate conformational sampling by a reverse-transformation from the CGMD level to the atomistic one. Atomistic simulations of back-mapped CGMD structures can recover the conformational properties of the original atomistic system. In this procedure, back-mapped atoms are randomly placed near their corresponding coarse-grained bead. The center of mass of these atoms is then restrained to the position of the coarse-grained bead. The system may be relaxed by a simulated annealing procedure to minimize large or unphysical forces, stochastically sample the conformation space and gradually introduce inter- and intra-molecular potentials that are consistent with the all-atom model. This method has been used to generate atomistic structures of simple peptides and transmembrane proteins from coarse-grained trajectories [162–164]. The back-mapping procedure also quantifies the information loss from coarse-graining, providing a useful way to validate a CG model against a more robust atomistic force field or extend a CG trajectory to include greater detail.

The earliest coarse-grained proteins were based on the Go model in which each amino acid is represented by a single bead that attracts or repels the other beads in the model according to interactions in the ground state. These models sought to investigate protein folding mechanisms [165]. Many of these models use non-standard molecular dynamics techniques. For example, discontinuous molecular dynamics was used to study the aggregation of peptides in implicit solvent [166,167], while Brownian dynamics simulations have been used to study crowding effects in the GroEL-GroES chaperonin system [168]. Elastic network models have found wide application in flexible fitting methods, which add detail to low resolution cryo-EM measurements [169]. Coarse-graining with empirical potentials is a common method for protein structure prediction and protein design [170,171].

Many of these coarse-grained approaches lack the chemical specificity necessary to study protein aggregation and association with lipid bilayers. The coarse-grained models described in Section 2 have been modified to include this detail in a number of ways.

There are many ways to generate intermolecular interactions for CG proteins. A common data set for generating non-bonded parameters in coarse-grained proteins is surface tension and density data for side-chain analogues. The surface tension characterizes the energetics of amino acid interactions at a vacuum interface; this quantity is a useful proxy for the attractive forces that mediate the interactions with water-lipid interfaces. The amino acid model developed by Klein and coworkers uses surface tension as target data and showed that the solvent accessible surface area (SASA) of the resulting protein models agreed with atomistic simulations [172]. The model was also able to recognize the native protein structures from a set of decoys. In another approach, Han *et al*. fit dihedral potentials for a test set of small molecules and tuned the force field to match self-solvation free energies and hydration free energies across a representative sample of organic molecules, finding good agreement to atomistic simulation [173]. In contrast, the model by Basdevant *et al.* [174] used a *r* ^6^ repulsive term with a Gaussian attractive term to represent non-bonded forces between amino acids, parameterized from atomistic simulation.

In an early extension of the Martini model [60] to proteins, Schulten and co-workers used residue-based coarse-graining (RBCG) as an intermediate scale in a multiscale model for membrane bending by Bin/Amphiphysin/Rvs (BAR) domains [105], described further in Section 4.2 . In this system, as well as applications to lipoprotein particles, the authors selected bead types from the Martini building blocks according to polarity and charge [6,175]. The authors also made minor modifications to protein bonded parameters to match atomistic simulations of their target systems [6]. Further coarse-graining by the shape-based coarse-graining method (SBCG) extended the model further, but discarded electrostatics, modeling lipids with only three beads and describing proteins with an elastic network model derived via iterative Boltzmann inversion [105,106,176].

Coarse-grained simulations developed by Voth and co-workers employs a Hybrid Analytical Systematic (HAS) model parameterized according to the MS-CG algorithm described in Section 2.3. The HAS model is based on the Gay-Berne ellipsoid particle model, which allows a single bead to represent a lipid. Lipid-protein interactions are modeled with a single Lennard-Jones term, and electrostatics are modeled with exponential screening according to Debye-Huckel theory, which is used to recover the polarizability lost during coarse-graining. A recent application of this model to N-BAR proteins tuned Lennard-Jones parameters for interactions between the membrane and amphipathic helix to match the atomistic peptide folding free-energy and empirical binding calculations [56,177,178]. Other recent modeling efforts have been extended to include DNA and RNA [179].

#### 3.2.2. Martini Proteins

**Figure 3:**
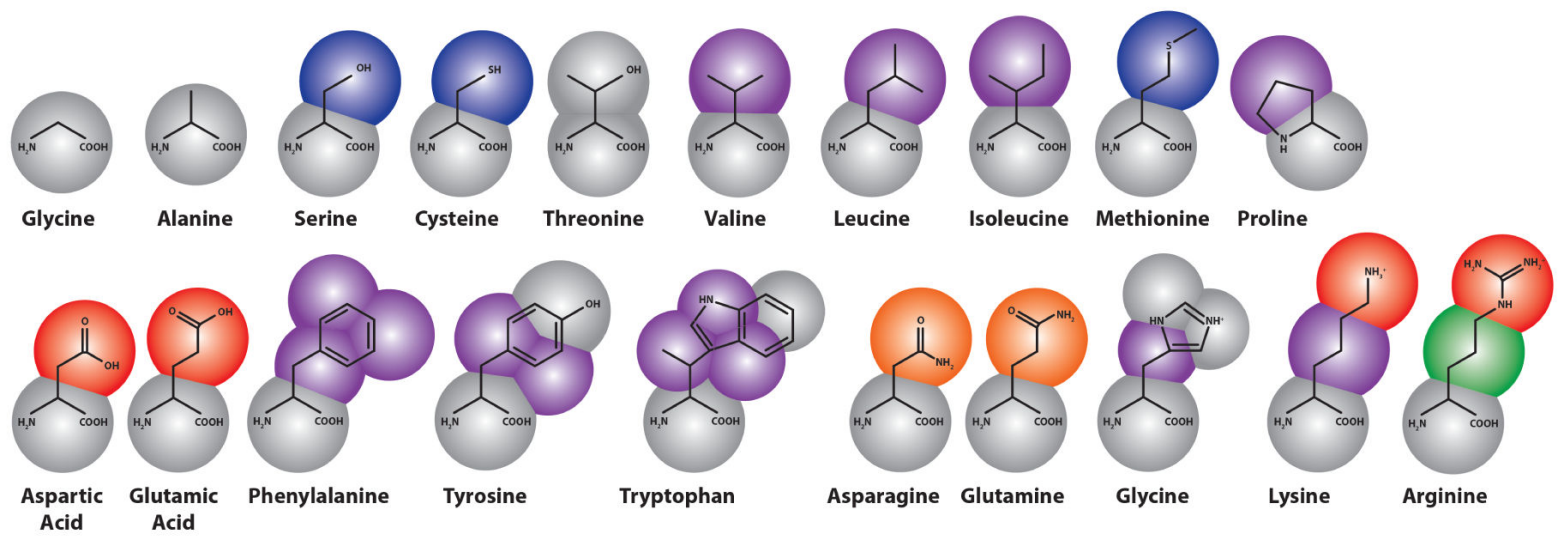
Coarse-grained representation of the Martini model extension to amino acids [181], colored by bead type (where purple is apolar, blue and green are intermediate, gray and orange are polar and red represents charged particles).

In the Martini force field, amino acids are mapped onto as many as five beads (see Figure 3), one of which represents the polypeptide backbone. Residues with rings (His, Phe, Tyr, Trp) use a finer mapping and improper dihedral terms to preserve the topology of these rings. Intra-amino acid bonded potentials—that is, bonds, angles and dihedrals—have equilibrium values equal to the average of distributions measured from all bonded amino acid pairs found in a representative sample of 2000 proteins from the protein data bank (PDB). These were sorted by helix, coil and extended secondary structure, as measured by the DSSP (“define secondary structure of proteins”) prediction algorithm [180], so that the Martini model includes the effect of secondary structure on the apparent hydrophobicity and polarity of its constituent particles. This secondary structure remains fixed through the simulation; therefore, the Martini model cannot sample secondary structure changes. However, it is possible to reconstitute atomistic details from a coarse-grained simulation using a “back-mapping” procedure similar to simulated annealing. This method has been demonstrated on simulations of the WALP transmembrane protein [163].

Both protein-protein and protein-lipid interactions are modulated by the non-bonded parameters, which are assigned via selection of side-chain bead types. This procedure has several parts. First, the bead types must partition between oil and water phases consistent with experiments in which the distribution coefficients of amino-acid analogs were calculated with NMR [182] and dynamic vapor pressure measurements [183]. These experiments are similar to those used to generate the partitioning free energies of the Martini alkane building blocks. In this case, a free energy perturbation method (FEP), called thermodynamic integration, was used to provide a more sophisticated measure of the *ΔG^oil=aqueous^* for each side-chain analog. In thermodynamic integration, a coupling parameter, *λ*, weights the addition of a single particle to the Hamiltonian. The derivative of this Hamiltonian can be numerically integrated to obtain the free energy difference for adding that particle. In this case, the partitioning coefficient is calculated from thermodynamic integration of the addition of side chain analogs to water and decane boxes [184]. A modification to the Bennett acceptance ratio method was used to calculate the free energy difference and associated errors [185].

A second validation of the protein force field is given by the potential of mean force (PMF) of the amino acid interactions with a lipid bilayer. The potential of mean force quantifies the free energy landscape according to a fixed coordinate, in this case, given by the distance of the amino acid from the center of the bilayer. Umbrella sampling and the weighted histogram analysis method (WHAM) [186] were used to generate the PMFs for comparison to atomistic PMFs calculated by MacCallum *et al.* using the OPLS protein force field [187].

It is generally difficult to compute a PMF analog from experiments, especially for peptides. However, a recent study calculated the PMFs of penta-peptides of the form Ac-WLXLL (where X is any one of the twenty natural amino acids). The free energies of partitioning of the variable residue were calculated using a thermodynamic cycle, which included the free energy change of displacement from the membrane via umbrella sampling and the alchemical introduction of the particle via thermodynamic integration [188]. These values were consistent with a measure of hydrophobicity called the Wimley-White scale, which groups amino acids into five categories according to their partition coefficients, as measured by a combination of equilibrium dialysis and quantitative reverse-phase HPLC for peptide hydrophobicity at palmitoyloleoylphosphatidylcholine (POPC) bilayers [189].

In addition to reproducing the correct association with lipid bilayers, proteins must associate with themselves in a physical way. To that end, association constants given by $K_{ij}=\frac{1}{C}\times \frac{P_{\mathrm{bond}}}{P_{\mathrm{free}}}$, where *C* is a concentration correction and the *P_bond_* and *P_free_* are the probabilities of finding a pair in a given bound or unbound state [184]. These were distinguished by a solvent accessible surface area (SASA) calculation, in which areas below a particular threshold indicate that the residues are contacting. The dimerization free energy was also computed directly from Equation (21), according to a radial distribution function given by the PMF over the distance between side chains [184].

(21) $$\Delta G^{\dim}=-k_{B}T\ln\frac{4\pi R_{\max}^{3}\int_{0}^{r_{c}}r^{2}g(r)dr}{3v^{\phi }\int_{r_{c}}^{R_{\max}}r^{2}g(r)dr}$$

In this equation, *g*(*r*) = *e*^*−PMF*(*r*)*=k*_*B*_*T*^, *R_max_* is the maximum distance between monomers, *r_c_* is the dimer-monomer cutoff distance, and *v^ϕ^* is the standard volume, 1 mol L ^1^. The dimerization free energy agrees with that measured in atomistic simulations in OPLS and GROMOS in test systems of amino acid pairs [190]. The dimerization free energy cannot be measured directly by experiment; however, a host of knowledge-based potentials have been designed to quantify the *4G^dim^* by ranking the co-occurrence between pairs of amino acids in known protein structures [191]. While these results may be influenced by the presence of a hydrophobic environment inside the body of the protein, it nevertheless provides a useful benchmark.

Recent improvements to the Martini model’s protein parameters [184] have included refinements to the free energy methods described above, as well as slight changes to the bead types for non-charged polar residues. Additionally, the development of a polarizable Martini force field has made it possible to improve the polar, but neutral, Asn, Gln, Ser and Thr residues. The polarizable extension to the Martini model includes a fluctuating dipole resembling the Drude oscillator in which two partial charges are tethered to a polarized bead and interact via a Coulomb function only. The dipole momentum is adjusted via harmonic angle and distance potentials. The resulting model therefore includes orientational polarizability, which makes it possible to more accurately model electrostatic interactions, particularly in transmembrane pores and antimicrobial peptide applications [74].

**Figure 4:**
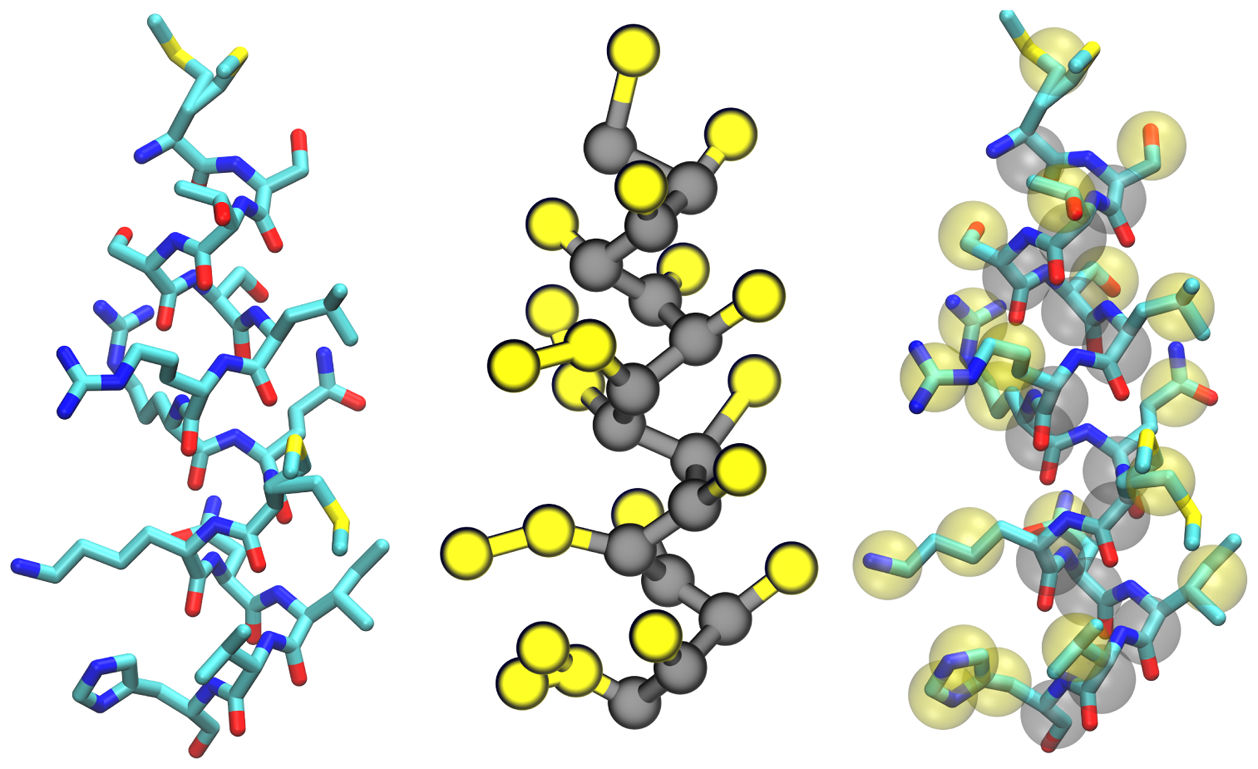
An example protein helix in all-atom (left) and Martini coarse-grained representations (center, backbone beads in gray and side-chain beads in yellow) with both images merged (right) to show how the fine-grained structure is mapped onto the coarse-grained beads. This image was rendered with Visual Molecular Dynamics [17].

In this section, we have described the ways in which the individual Martini building blocks were adapted to include the interactions between systems of proteins (see Figure 4 ) and lipids. Careful parameterization of these building blocks ensures that the model is capable of reproducing the complex behavior of many biomolecular systems. Applications to richer biological problems validate the model, while providing molecular insight into experiments, for example, an extension of the Martini protein force field to model the aggregation of amyloid-like peptides [192].

### 3.3. Improvements to Protein Models

While most of the efforts to incorporate proteins into coarse-grained simulations of soft matter has rightly focused on parameterizing the interactions of amino acids with water, lipids and each other, cutting edge development of more advanced force fields has explored the possibility of capturing conformational sampling in coarse-grained models. If CGMD can accurately explain protein-bilayer interactions, peptide self-assembly and protein binding, then, it is reasonable to see whether these methods can also model internal structural changes that guide the biological functions of many proteins. This is an extension of the previous challenge: to accurately capture peptide-peptide interactions and their relationship to the intra-molecular (bonded) forces, which makes the complex conformational equilibria of polypeptides possible.

## 4. Membrane-Protein Applications

In Sections 2 and 3 , we have shown how coarse-grained molecular dynamics simulations are constructed from chemical components, which match molecular experiments. We see that these models are capable of reproducing the fundamental properties of the systems they mimic, including protein structure and dynamics, peptide-bilayer interactions and the geometry and elasticity of membranes. In this section, we will show how these models can reproduce the behavior of far more complicated biophysical systems, yielding insight to experiments and elucidating the molecular mechanism by which proteins interact with cell membranes.

### 4.1. Simulations of Biological Membranes

While the parameterization of any soft matter CGMD force field includes validation of membrane fluidity and geometry, these models must also capture the condensed matter properties of biological membranes. Any simulation that seeks to quantify protein-mediated membrane properties must be capable of mimicking the properties of a bare membrane.

The earliest Martini model simulations sought to reproduce the complex phase behavior of bio-mimetic membranes. Simulations of phosphatidylcholines of differing lengths separated into gel and liquid phases in a small temperature range and semi-quantitatively matched experimental phase transitions, which were modulated by their relative concentrations [193]. Formation of non-lamellar phases is essential for modeling the first steps of membrane fusion and can be induced in Martini bilayers by varying temperature and hydration levels. Simulations of mixed dioleoylphosphatidylcholine with dioleoylphosphatidylethanolamine (DOPC/DOPE) bilayers mimicked hexagonal, inverted hexagonal and rhombohedral phases according to X-ray diffraction experiments [194,195] with precise control of hydration levels [196]. Further tests have studied the temperature dependence of the the fluid-gel phase transition for DPPC bilayers, providing thermodynamic parameters, namely, estimates for the line tension and entropy difference of the fluid-gel interface [197]. These measurements connect simulations with non-equilibrium experiments using X-ray diffraction with pressure-jump relaxation [198] and temperature scanning calorimetric, densitometric and acoustic measurements, providing insight into the kinetics of these phase transitions [83]. A study of the effects of lipid compositions identified lipids with varying levels of saturation that lower the line tension at domain interfaces in the bilayer [86,87,199].

Having established that coarse-grained bilayers exhibit the phase behavior features of bilayers observed in experiments, researchers began to study peptide-bilayer interactions. Simulations of the influenza HA fusion peptide revealed a bi-continuous cubic phase by stabilizing stalk/pore complexes in agreement with *in vitro* measurements that show that the peptide lowers the lamellar-to-inverted hexagonal phase transition temperature [10]. Simulations of antimicrobial peptides show that they adhere to bilayers, assemble into amphipathic nanotubes and extrude lipids from the bilayer [200]. Antimicrobial peptide aggregates also induce long-range order in phosphatidylglycerol domains, in agreement with atomic force and TIRFexperiments [201].

Having demonstrated that the Martini model accurately predicts these phase transitions, these models have been extended to systems that simulate vesicle fusion. Initial studies of vesicle fusion events show a branched pathway for fusion in which stalk-like structures may either form a fusion pore or slowly fuse via a hemi-fused state [202,203], and subsequent study estimates the free energy barrier to fusion and show that the kinetics of the early stages of fusion are determined by the energy of solvent-exposed lipid tails [204]. Simulations of lung surfactant protein show the mechanism by which they facilitate the formation of a lipid bridge in vesicle fusion [11,85,205]. Likewise, monolayer simulations in the Martini model [84,85] and a recent coarse-grained force field by Shinoda *et al.* [69] have been used to explore monolayer buckling. In addition to coarse-grained MD, a mesoscale method called dissipative particle dynamics can be used to study vesicle fusion [12]. Mixtures of double-stranded DNA and lipids, called lipoplexes, are potential transfection vectors that have a lower toxicity than viral vectors. Martini simulations have matched observations from SAXS and other experiments, which observed a lamellar to inverse-hexagonal phase transition [205,206].

Lipid rafts have been known to provide additional compartmentalization of the cell membrane, serving to organize and direct the action of biomolecular complexes. CGMD simulations have shown that thickness mismatches between phases are communicated to opposing leaflets and assist in guiding rafts together or stabilizing a registered geometry [207]. Martini simulations show broad agreement with NMR measurements [208], which show that cholesterol preference for saturated tails drives phase separation.

Finally, simulations of biological membranes are not limited to models that include explicit solvent particles. For example, Deserno and co-workers have applied a solvent-free coarse-grained model with three beads per lipid [209] to study membrane remodeling by generic viral capsids or colloids, finding that such particles attract, due to their induced membrane curvature [210].

A generic implicit model by Brown and co-workers has been used to study the effects of protein inclusions in lipid bilayers [211,212]. Likewise, a solvent-free combination of the MS-CG method with short-ranged coarse-grained potentials has been used to simulate liposomes [178]. Recent efforts have tuned implicit coarse-grained bilayer models to reproduce bilayer stress profiles [213]. The range of coarse-grained approaches for simulating lipid bilayers makes it possible for researchers to select the desirable level of detail necessary to study a biological system of interest. In the next section, we will review one such example in which protein-protein and protein-membrane interactions at different length scales act in concert to remodel the membrane.

### 4.2. Modeling Membrane Bending

Membrane remodeling by Bin/Amphiphysin/Rvs (BAR) domains provides an archetypical application of the combined soft-matter and protein coarse-graining methods described above [2,105,106]. Highly conserved, ubiquitously expressed BAR domains bend cellular membranes from the cytosol, participating in endocytosis, vesicle fusion, cell-cell fusion and, also, apoptosis. *In vivo*, members of the BAR domain, such as N-BAR, form high-curvature tubes with a low radius of (~50 nm), while *in vitro*, they form vesicles from liposomes. N-BAR includes an N-terminal amphipathic helix (helix-0), which may either scaffold the charged lipids that contact it or induce an area asymmetry (or both) in service of bending the membrane.

**Figure 5:**
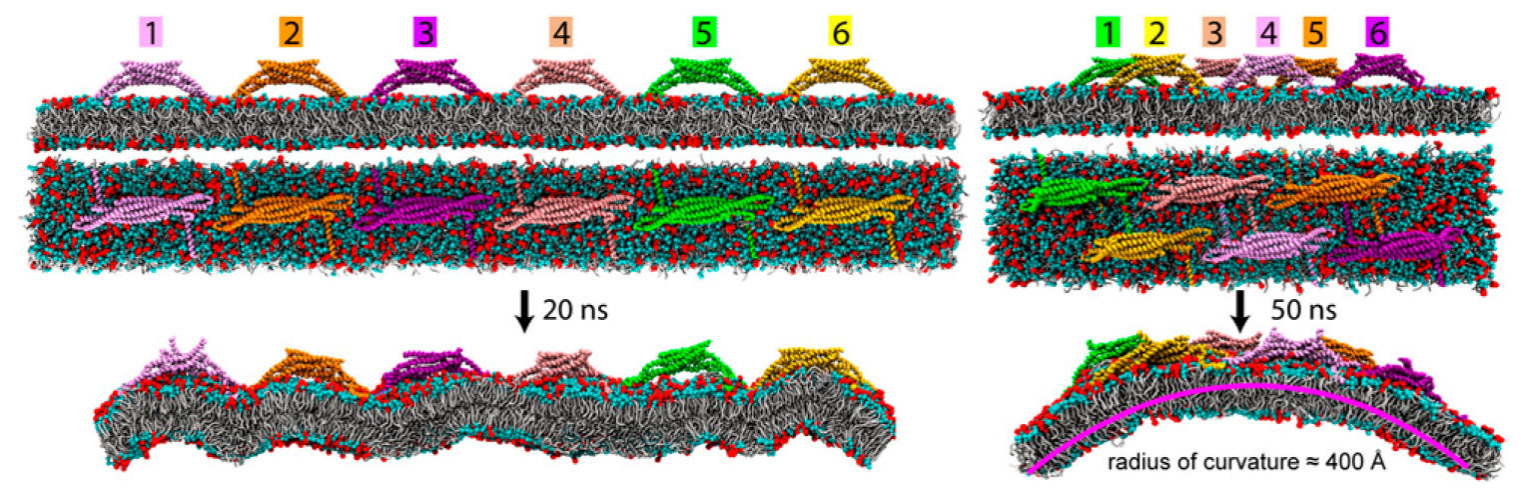
Simulations of six Bin/Amphiphysin/Rvs (BAR) domains remodeling a membrane. These simulations show that the proteins require a staggered arrangement (right) to bend the membrane, while the non-staggered arrangement (left) fails to generate curvature. Figure adapted from Arkhipov, *et al.* [105].

All-atom molecular dynamics simulations of N-BAR bending a 7:3 dioleoylphosphatidylcholine with dioleoylphospatidylserine (DOPC/DOPS) membrane showed that the protein stabilized local curvature [2,105,106], see Figure 5 . These simulations were used to quantify the flexibility and tertiary structure of a single domain. These results were used to construct bonds between coarse-grained beads via Boltzmann inversion in the so-called residue-based coarse-graining (RBCG) method, which uses non-bonded forces adapted from the original Martini lipid force field [6,105,176]. A low dielectric constant (*ε* = 1) was necessary to reproduce the strong electrostatic contacts responsible for bending the membrane to adhere to the N-BAR surface.

The RBCG simulations tested staggered and ordered arrangements of six N-BAR domains on a membrane patch, finding that only the former yielded a stable, global bending mode. Further coarse-graining under the shape-based (SBCG) method, in which lipids of ~150 atoms are represented by two beads connected by a harmonic spring, extended these simulations to still larger time scales (5 μs). Non-bonded LJ parameters in this model were tuned to reproduce area-per-lipid and bilayer thickness measurements. The SBCG simulations also provided an estimate of the membrane bending modulus by measuring the force exerted on the edges of a bilayer tube, confirming that the SBCG N-BAR domains have sufficient energy to bend the membrane. To explore the dynamics of membrane bending, the measured curvature from SBCG provided the intrinsic curvature parameter for a continuum elastic membrane model, which included membrane bending, stretching and viscous drag forces [105]. Testing the sensitivity of these parameters showed that drag forces determine the damping of the remodeled membrane. This study matched the structure seen in cryo-TEM images of the tubules, which failed to locate the precise ordering and orientation of the four inserted amphipathic helices, but found that helix-0 interactions are degenerate, dynamic and necessary for stabilizing the lattice [177]. In the absence of dimers of amphipathic helices, BAR domain oligomers show reduced orientational order and cannot form a stable lattice. CGMD simulations of liposomes shows that N-BAR forms a lattice that is both higher order and higher density, suggesting that this density is necessary to induce high, stable curvature *in vivo* [214].

In a related system, the protein, epsin, is hypothesized to sense and induce curvature, while recruiting accessory proteins in the early stages of clathrin-mediated endocytosis (CME). The highly-conserved epsin N-terminal homology domain (ENTH) binds phosphatidylinositol 4,5-bisphosphate (PIP_2_) by inserting an N-terminal amphipathic helix similar to that found in the BAR domains (helix-0). This helix becomes helical upon membrane binding according to circular dichroism, and ENTH domains were found to tubulate liposomes *in vivo* [215].

According to spin-labeled electron paramagnetic resonance (EPR) spectroscopy measurements, helix-0 becomes structured when binding the PIP_2_ headgroup [216]. Further EPR studies and AAMD simulation provides a more detailed description of helix-0 penetration and key distances between ENTH domains, and these parameters were integrated into a CGMD model for ENTH-induced tubulation [217]. This model showed that heterogeneous lattice reduces the anisotropy of the spontaneous curvature and tends to frustrate tubule formation, thus explaining experimental observations that high initial concentration of ENTH domains tends to form vesicles (which have isotropic curvature by definition), while adding ENTH to preformed membrane tubules crystallizes proteins in a more ordered, helical pattern [217]. It is important to note that the protein, epsin, and the family of BAR domains are only two examples of a diverse set of membrane remodeling proteins. Other proteins have been shown to induce curvature, as well, likely via different mechanisms. For example, the protein, *α*-synuclein, the protein implicated in Parkinson’s disease, induces negative Gaussian curvature according to coarse-grained molecular dynamics simulations, which were then matched to low-angle X-ray scattering data, which highlight the thinning effect on the bilayer [218]. The mechanism of protein-induced curvature sensing and generation has been recently reviewed by Baumgart *et al.* [219].

### 4.3. Lipid Bilayers Support Protein Assembly and Function

While many proteins actively remodel bilayers during biological processes, in contrasting mechanisms, the bilayer (or liquid-liquid interface) provides a substrate for protein assembly. The CMM-CG model developed by Klein and coworkers found early application in modeling the interaction of synthetic hydraphiles with bilayers [220] and the assembly of peptide nanotubes at oil-water interfaces [7]. Transmembrane peptides demonstrate the ability to sort lipids by chain length when they are smaller than the bilayer thickness [221,222]. These simulations show that the peptide induces a meniscus, which depletes water from the peptide and encourages bilayer fusion, thus explaining experiments that show that these peptides can induce a transition from the lamellar to inverted phase. The protein force field extension by DeVane *et al.* was used to simulate the behavior of hydrophobins, proteins that self-assemble at air-water interfaces [223], in agreement with experimental measurements of adsorption and desorption free energies of comparable molecules. Tilt angles and helix-helix association of transmembrane peptides modeled with the Martini force field agreed with those measured from solid-state NMR [8,181]. Lateral diffusion rates measured by fluorescence correlation spectroscopy (on confocal laser-scanning microscopy) quantify the diffusion rate of transmembrane proteins in bilayers; this rate is modulated by membrane thickness and composition, with little effect from the lipid headgroup [224].

The structure of the HIV-1 virion is another valuable candidate for coarse-grained study, because it shows a relatively complex morphology that is generated from the components of a single polypeptide, about which much is known. In a recent multiscale simulation, Ayton and Voth used CGMD to reproduce structural features of the virion as observed by electron microscopy and cryotomography. Noting that only enhanced interactions the C-terminal capsid domain were sufficient to stabilize the hexameric lattice on the immature virion, they performed AAMD simulations to validate the CGMD model, showing that close-contact sites have a PMF well that is twice as deep in the wild-type compared to mutants, which show reduced viral infectivity in cells and particle defects under transmission EM [225]. Coarse-grained simulations and PMF calculations of lipid-mediated protein interactions have also been used to study the hydrophilic shielding of proteins within a bilayer [226,227].

In an advanced application, CGMD has been applied to the study of protein-gated ion channels. Simulations of the plug domain in SecY shows that the introduction of a disulfide bond is sufficient to open the channel, explaining unrestricted translocation seen in experiments using a disulfide-immobilized plug domain [4]. Simulations of the mechano-sensitive protein channel, MscL, characterize the decrease in liposome stress as the channel activates [107]. Simulations of voltage-gated potassium (Kv) channels have characterized the closed structure, while matching experimental constraints [228], including pore radius measurements, electrophysiology observations and histidine scanning. Simulations have also investigated carbon nanotube-lipid interactions [229,230], confinement of copolymers [231] and pore formation by dendrimers [232–234].

The class of G-protein coupled receptors (GPCRs) represents another relevant protein-membrane mechanism. Martini simulations of a particular GPCR, rhodopsin, show that it self-assembles via a hydrophobic mismatch mechanism by matching simulations to EPR and FRETexperiments with bilayers of varying thickness [235]. Free energy profiles calculated from Martini simulations of glycophorin A (GpA) show that mutations to this transmembrane alpha-helical protein disrupt its association in bilayers, but do not abolish it, suggesting that non-specific aggregates are possible [8,13,236,237]. These results agree with experiments that quantify the self-assembly of GpA using sedimentation equilibrium analytical centrifugation, FRET and thiol disulfide interchange experiments [238]. Studies of high-density lipoprotein “nanodiscs” self-assembled on bilayers and matched temperature-dependent swelling of the particle observed in SAXS measurements and hydrophobic mismatch at the protein-lipid interface observed via solid state NMR [6,175,239].

In addition to the multiscale model for membrane bending by BAR domain proteins described in Section 4.2 , there has been considerable effort to connect atomistic, coarse-grained and mesoscale models to study other protein-membrane systems. For example, a hybrid molecular mechanics/ coarse-grained (MM/CG) model has been used to simultaneously improve the resolution of coarse-grained systems and extend atomistic ones to larger scales. In this approach, soft boundary potentials divide the atomistic and coarse-grained representations with an overlapping interface region. These simulations include stochastic and frictional forces, due to the solvent, along with cross-potentials designed to distribute coarse-grained forces across their constituent atoms in the interface region. The hybrid approach retains key microscopic details, including hydrogen bond networks and the root mean squared fluctuations (RMSF) of the protein structure. It has found application in the study of enzyme active sites [240] and outer membrane proteases [241]. The hybrid approach has also made it possible to describe the ligand binding site for GPCRs, in good agreement with atomistic simulation, suggesting a future role for this method in drug design [242]. Other hybrid approaches merge atomistic models with continuum methods in order to study protein-nucleic acid complexes [243] and membrane-peptide association [244]. Finally, hybrid models, which use virtual sites in the interface region, have been developed to bridge the Martini model with atomistic force fields [245]. The development of these hybrid modeling strategies makes it possible to customize coarse-grained simulations to include atomistic detail when necessary, broadening the range of possible applications to include specific protein-ligand binding. The hybrid approach further improves the flow of information between coarse-grained and atomistic representations by explicitly merging both models in the same simulation.

### 4.4. Extending to the Mesoscale

In much the same way that atomistic simulations inform coarse-grained models (and *vice versa*), coarse-grained models also make contact with mesoscale continuum mechanics models. Common to many of the membrane applications is the use of the Helfrich Hamiltonian [96], given by Equation (22), in which the membrane energy, *H*_el_, is modeled as an infinitesimally thin elastic sheet.

(22) $$H_{\text{el}}=\int \left\{\frac{\kappa }{2}{\left(H-H_{0}\right)}^{2}+{\bar{\kappa }}_{G}G+\sigma \right\}\;dA$$

Equation (22) is integrated over the surface area, *A*, and consists of terms that account for surface tension, *σ*, as well as bending and Gaussian curvature, with energies given by their respective bending rigidities, *κ* and *κ̄_G_*. In this formulation, the mean curvature, *H* = *c*_1_ + *c*_2_, and Gaussian curvature, *K* = *c*_1_*c*_2_, where *c*_1_ and *c*_2_ are the principal radii of curvature. Membrane remodeling enters the Helfrich in two places: via the spontaneous (or intrinsic) curvature term, *H*_0_, and also by modulating the bending rigidity of the underlying membrane.

Theoretical study of the Helfrich model has been used to explain vesicle configurations [246] and the effects of undulations on membrane elasticity [247,248]. Protein-induced deformations have been added to these models, which can then make contact with CGMD simulations. For example, the theory predicts an elastic response to cylindrical protein inclusions, which can be matched to ion channel experiments and further resolved with CGMD [211,212,249,250]. The Helfrich model may also be coupled to mesoscopic solvent models in order to include the effects of hydrodynamics on membrane motion [251].

While modeling protein assembly on lipid bilayers is a prime candidate for coarse-grained simulation, continuum methods often augment CGMD simulations. For example, multiscale study of the BAR domain proteins uses coarse-grained simulations as a bridge to continuum methods to understand the time-scales required for membrane bending by N-BAR [105]. A host of coupling algorithms may be used to bridge the gap between atomistic simulation and continuum methods. High resolution simulations provide the chemical detail, while numerical methods make it possible to apply them to realistic models [252,253].

Continuum mechanics simulations do not necessarily require coupling to atomistic or coarse-grained simulation, however. There is much to be learned from minimal mesoscale models that make contact with experiments. There are chemical and mechanical similarities between many membrane remodeling proteins [219]. Minimal mesoscale models can resolve the partitioning behavior of curvature-inducing proteins and the energetics of bud formation [254–256]. By modifying the direction, strength and anisotropy of the spontaneous curvature induced by a particular membrane-remodeling protein, these models can predict the geometry and energetics of the resulting cellular morphologies. The wide range of continuum methods described in this section suggest that mesoscale simulation and coarse-grained simulations may be employed together or separately to characterize membrane-remodeling events.

## 5. Conclusions and Future Directions

This review has surveyed the ways in which coarse-grained molecular dynamics simulations provide a crucial bridge between the chemical detail found in atomistic simulations of membrane proteins and the biologically relevant time- and length-scales accessible by continuum methods. Coarse-grained molecular dynamics simulations are ideally suited to simulating soft-matter systems relevant to biology, because they are efficient enough to represent diverse cellular morphologies, but descriptive enough to distinguish the energetics and geometry of systems with different lipid compositions and, amazingly, differences in protein sequence and structure.

As is evidenced by the competing methods for designing and tuning coarse-grained force fields, there is no single coarse-grained method that can produce the same description as an atomistic one. The choice between structure, thermodynamic and force-matching coarse-graining strategies depends strongly on the system of interest, computational resources, target experimental data and, most importantly, the question that the model must answer. Though many of these methods are able to reproduce some combination of structural and thermodynamic data, there is no guarantee that a naive coarse-grained simulation will produce accurate results. Careful matching to theory, simulation and experiments ensures that a particular model is physically accurate. More importantly, contact between these methods provides perspective on the physics of biological processes. In particular, we see that lipid bilayers mediate a host of cell processes, from the action of mechanically-sensitive ion channels, to morphology-generating membrane-remodeling, to the activation of complex cell-signaling networks by membrane-associated proteins. Future study of protein-membrane systems with coarse-grained methods will depend on synthesizing our understanding of soft matter systems with biology and biochemistry. This field of study has the potential to improve human health by resolving cell biological process at high resolution and, moreover, guiding the design of new treatment strategies.

## Figures and Tables

**Table 1:** Summary of key modeling calculations and target data for representative coarse-grained models discussed in Sections 2.2 –2.4. This list is not exhaustive, however, and these models reproduce a wide range of experimental data.

Model	Key Methods	Key Target Data
CMM-CG [44,69]	structure matching, energy matching, Boltzmann inversion, reverse Monte Carlo	density distributions, interfacial tension, area per lipid, bending modulus, area compressibility modulus, lipid order parameters
MS-CG [49,55]	bottom-up force matching, variational optimization, cubic spline basis functions, hybrid analytic-systematic coarse-graining, screened electrostatics	atomistic site-to-site radial distribution functions, density distributions, bending modulus, area compressibility modulus, lipid diffusion rates
Martini [16,60]	top-down energy matching, potential of mean force between phases, bilayer stress profile, free energy of lipid desorption or flip-flop, short-range electrostatics	free energy of hydration, free energy of vaporization, partitioning free energies, surface tension, interfacial tension, density distributions, bending modulus, area per lipid

**Table 2:** Summary of corresponding experimental methods and simulation measurements which may be used match key physical properties of soft matter systems. GUV, giant unilamellar vesicle.

Property	Experimental Method	Simulation Measurement
partition coefficient	titration calorimetry	potential of mean force of a particle pulled between phases
self-diffusion coefficient	magnetic resonance spin echo	mean-squared displacement
electron density profile	X-ray scattering	electron density
area per lipid	neutron scattering	area measurement (bilayer mid-plane)
lipid order parameter	nuclear magnetic resonance (NMR)	lipid tail angles to the bilayer normal
phase transition temperature	cryo-transmission electron microscopy (cryo-TEM)	structure factor
pressure-area isotherm	Langmuir trough, captive bubble surfactometer	pressure tensor, area measurement
line tension	fluorescence microscopy of GUVs, micropipette aspiration	pressure tensor height-height fluctuation
bending rigidity	video phase contrast microscopy, GUV shear flow	spectrum
